# Round the Hospitals

**Published:** 1917-05-05

**Authors:** 


### ROUND THE HOSPITALS.

The "Kill the College" and "Divide British Nurses" party, which never fails to maintain that the direct representation of the great body of British nurses forms no part of the constitution of the College, may be glad to have the information as further and striking evidence to the contrary, that the following nurses have been elected members of the Scottish Board of the College: Miss Jessie Henderson and Miss Catherine Gardner, Nurses' Co-operation, Glasgow; Miss Ruby McPhail, Edinburgh; Miss Catherine Kay, Nursing Home, Edinburgh; Miss Morrison and Miss Flora Ross, of the Queen Victoria Jubilee Nurses' Institute.

A grand concert for nurses, arranged and provided by Mr. Victor Beigel, at which Her Majesty Queen Alexandra and H.R.H. Princess Christian were present, was held on the 2nd instant at the Queen's Hall, Langham Place, under the presidency of the Hon. Arthur Stanley, C.B., M.P., chairman of the College of Nursing (see pp. 95 and 96). The noble effort of the British Women's Hospital to raise the Nation's Fund for Nurses to provide an Endowment Fund for the College and funds out of which provision can be made for those members of the nursing profession who through no fault of their own are in need or in suffering, will constitute a tribute from the British Empire to the British nurses, and cannot fail to win the support of all classes of people. Who is there who will not welcome an opportunity by an offering to this Fund to do their best to repay to the nurses themselves some part of the vast debt owed to them by the community to which they have ministered so devotedly? The Nation's Fund for Nurses, its objects and scope are explained on page 93.

Great interest was taken by the nurses present at a meeting held in the Nurses' Recreation Room of the Chelsea Infirmary on April 27, when Miss Rundle gave an address on the College of Nursing, full of practical points. One great point was the individual responsibility of each fully trained nurse, both before and after she becomes a member of the College, because its establishment on the lines most useful to the profession, can be best secured by the knowledgeable interest of its members. The election of the Council after the first two years will mainly be in the hands of the nurse members. It is the privilege and duty of each nurse to educate and equip herself to discharge this responsibility impartially and with sound judgment. The co-operation of the members possessing this knowledgable interest will be required increasingly to help solve the many problems with which the College will be confronted. Miss Redl, matron of the Brompton Hospital, and some of her staff were present at the meeting, as well as Miss West, matron of the Chelsea Hospital for Women, and many members of the staff of the Chelsea Infirmary. An interesting discussion arose which was full of promise, for discussion at such meetings should be the life-blood of the College.

At the quarterly meeting of the Poor-Law Infirmary Matrons' Association, held on April 28, at the Kensington Infirmary, an interesting discussion took place as to how the Association could best benefit the new associate members, i.e., the superintendent nurses of the smaller infirmaries. Miss Bodley, matron of Selly Oak Infirmary, Birmingham, read a paper suggesting various ways in which the Association could help Poor-Law trained nurses. Of them, the first in importance no doubt is to so add to the scope of the P.L.I.M.A. as to organise a society of all fully trained Poor-Law infirmary and other nurses so as to give them the authority, strength and power to uphold their status and independence. This is a most urgent and pressing matter, for it would knit together all certificated nurses in the service, and so secure them the position which is their due. Seeing that the rules of the Association are to be amended, in view of its future expansion, and that all past members are to be invited to become honorary members of the Association, it is wise to go a step further, and bring all fully trained Poor-Law nurses into direct co-operation with the P.L.I.M.A.

The Army's demand for fully trained nurses is now almost as urgent as its demand for doctors. Following upon the Minister of War's appeal, a statement has been made by the Matron-in-Chief at Adastral House which puts the position very clearly. "All retired nurses who are free to serve," says the Matron-in-Chief, "should arrange to place their services promptly at the disposal of civil institutions at home. It is impossible as yet to say how many will be needed to complete the new hospital establishments, but there is no doubt that the demand for a great many more nurses is most urgent. We want nurses who are ready to work wherever required, at home or abroad. There is ample scope for medical as well as surgical nurses." The Matron-in-Chief adds that applications by nurses holding three-year certificates will be promptly dealt with. To begin with, nurses may sign for a year, or, in special cases, even a six months' agreement may be arranged. Longer service brings an increased rate of pay.

